# Adaptation of child oral health education leaflets for Arabic migrants in Australia: a qualitative study

**DOI:** 10.1186/s12903-017-0469-z

**Published:** 2018-01-10

**Authors:** Amit Arora, Ibrahim Al-Salti, Hussam Murad, Quang Tran, Rhonda Itaoui, Sameer Bhole, Shilpi Ajwani, Charlotte Jones, Narendar Manohar

**Affiliations:** 1 School of Science and Health, Western Sydney University, 24.2.97 Campbelltown Campus, Locked Bag 1797, Penrith, NSW 2751 Australia; 2 Oral Health Services and Sydney Dental Hospital, Sydney Local Health District, Surry Hills, Australia; 3 Discipline of Paediatrics and Child Health, Sydney Medical School, Westmead, Australia; 4 Collaboration for Oral Health Outcomes Research, Translation, and Evaluation (COHORTE) Research Group, Ingham Institute for Applied Medical Research, Liverpool, Australia; 5 Faculty of Dentistry, The University of Sydney, Westmead, Australia; 6 School of Social Sciences and Psychology, Western Sydney University, Penrith, Australia; 7 Faculty of Medicine, University of British Columbia, Vancouver, Canada

**Keywords:** Culturally and linguistically diverse, Arabic, Oral health, Migrant, Australia

## Abstract

**Background:**

The purpose of this study was to gain an in-depth understanding of Arabic-speaking mothers views on the usefulness of existing oral health education leaflets aimed at young children and also to record their views on the tailored versions of these leaflets.

**Methods:**

This qualitative study was nested within a large ongoing birth cohort study in South Western Sydney, Australia. Arabic-speaking mothers (*n* = 19) with young children were purposively selected and approached for a semi-structured interview. Two original English leaflets giving advice on young children’s oral health were sent to mother’s prior to the interview. On the day of interview, mothers were given simplified-English and Arabic versions of both the leaflets and were asked to compare the three versions. Interviews were audio-recorded, subsequently transcribed verbatim and analysed by thematic analysis. Ethical approval was obtained from Human Research Ethics Committees of the former Sydney South West Area Health Service, University of Sydney and Western Sydney University.

**Results:**

Mothers reported that simplified English together with the Arabic version of the leaflets were useful sources of information. Although many mothers favoured the simplified version over original English leaflets, the majority favoured the leaflets in Arabic. Ideally, a “dual Arabic - simplified English leaflet” was preferred. The understanding of key health messages was optimised through a simple layout and visual images.

**Conclusions:**

There is a need to tailor oral health education leaflets for Arabic-speaking migrants. Producers of dental leaflets should also consider a “dual Arabic – simplified English leaflet” to improve oral health knowledge of Arabic-speaking migrants. The use of simple layout and pictures assists Arabic-speaking migrants to understand the content of dental leaflets.

## Background

Early Childhood Caries (ECC) is a significant health problem worldwide [1]. The prevalence of dental caries among Australian children has been on the rise since mid-1990s [2]. The most recent Australian Child Oral Health Survey (2012–2014) reported that nearly 25% of 5 to 10-year-old children had untreated dental caries in the primary dentition, while one in ten children aged 6 to 14-years had untreated caries in the permanent dentition [3]. Factors associated with the development and progression of dental caries are complex and may include an interplay between genetic inheritance, socio-demographics, lack of access to dental care, poor dental care utilisation, low health literacy, beliefs; inappropriate health behaviours, cultural beliefs and practices and bacterial infection (predominantly mutans streptococci) [4]. Despite of such complex causal characteristics, dental caries in children can be prevented by providing health promotion to the families [5] to ensure early adoption of healthy behaviours.

The health promotion messages to maintain good oral health are well documented [5, 6] and can be beneficial if adopted early in life and become a part of routine life. The key oral health promotion messages include: brushing twice daily with fluoridated toothpaste, reducing the frequency of sugar consumption and seeking regular visits to an oral health professional. There is consensus that people who have appropriate health knowledge and skills are more likely to improve their quality of life [7, 8]. However, evidence suggests that health-related behaviours are strongly influenced by social norms [9], which are related to education, social class, and ethnicity [10].

Changing health behaviours is complex but is largely dependent on accessing, reading, understanding, and processing health information [11]. In developed countries during the 1970s, health education initiatives were aimed at behaviour change to prevent chronic diseases [12]. Most initiatives were heavily reliant on simple understanding that the transmission of health information will lead to behaviour change [13]. Over time, it became evident that health education may increase health knowledge but was not enough for sustained behaviour change [5]. This concept was strengthened in the 1980s, during which several theories were proposed on how social and personal skills influence decisions related to health behaviours. This helped to explain the relationship between knowledge, beliefs and social norms; and provided useful insights on the content of health promotion programmes [13].

In dentistry, leaflets have been the traditional method of conveying health information to consumers. However, there is overwhelming evidence that the value of these health education resources will be compromised by an individual’s literacy skills [7, 14–17]. The inability to read, understand and act-upon health education messages may contribute to health inequality, and this is certainly true in dentistry aspect where children from ethnic minorities are more likely to have dental health problems due to lower levels of literacy, lack of understanding on how to access and utilise dental services; and inappropriate culturally-specific norms and beliefs [18, 19]. Hence, all health education initiatives aimed for ethnic minorities should take literacy and cultural appropriateness into account for raising oral health awareness. Despite the propensity to utilise leaflets as the key medium for oral health education, there remains limited empirical evidence on whether such medium addresses the needs of migrants living in disadvantaged areas of Australia, such as Greater Western Sydney.

The World Health Organization defines Health literacy as “the cognitive and social skills which determine the motivation and ability of individuals to gain access to, understand and use information in ways which promote and maintain good health” [20]. Lower levels of health literacy have been associated with less focus on disease prevention and higher health care costs [21–23]. In an oral health context, literacy skills are crucial for people to understand the factors influencing oral health, to adopt oral health promoting behaviours, to communicate with oral health professional team, or organise dental appointments, to find their way to the dental clinics, to fill out the necessary forms at the dental appointment, and to comply with any required regimes, including follow-up appointments and prescribed medications [24]. Ethnic minority groups are particularly susceptible to poor oral health literacy [15, 17], which in part, may be implicated in the observed oral health disparities. There is a paucity of research and knowledge around this issue, including how to overcome the barriers related to appropriate oral health literacy. Enhancing the oral health literacy of at-risk cultural minority groups living in multicultural countries such as Australia is fundamental to the alleviation of health disparities and overall disadvantage.

According to the Australian Bureau of Statistics (ABS), individuals residing in rural areas and from a lower socio-economic backgrounds (lower levels of income, education and non-English-speaking) have lower levels of health literacy [25]. Indeed, recent migrants to Australia, having English as a second language may also have limited literacy in their native language to some extent [8]. Hence, this inability to access, engage-with and understand health education messages in both their first and second language might lead to compromised health among ethnic minority groups living in Australia [18, 19]. Furthermore, migrants, among other groups residing in disadvantaged areas, experience additional drawbacks such as a higher prevalence of health risk factors and, difficulty accessing and utilising health services in comparison to the more advantaged counterparts [26, 27].

To be specific, Arabic-speaking population is a fast growing minority population in Australia. According to the ABS Census in 2011, the Arabic-speaking population represents 1.3% of the total Australian population [28]. The Arabic-speaking community in Greater Western Sydney forms 6.6% of its total population; with Arabic being the top non-English language spoken at home [29]. Across Australia, there is a great diversity amongst the Arabic-speaking community, as these families originate from over 22 countries where Arabic is either an official language or spoken by a significant portion of the population [30]. Furthermore, in the last 10 years, some people from Arabic-speaking countries have migrated to Australia as refugees or asylum seekers [8, 28, 31]. Due to this migration experience, a proportion of migrant Arabic-speaking population have significantly lower incomes, lower levels of English proficiency, and education compared to the local Australians or other major migrant populations [32]. As a result, they are particularly susceptible to poor oral health literacy and difficulty accessing the local health care services. Furthermore, the majority of Arabic-speaking community in Greater Western Sydney resides within a socio-economically disadvantaged geographical location [33].

Hence, based on the socio-economic and geographic disadvantage faced by Arabic-speaking migrants in Greater Western Sydney, compounded by English language barriers, the need to assess the extent to which existing oral health leaflets address the oral health literacy needs of Arabic migrants is exemplified. Thus, the aims of this study were two-fold: First, to explore the views of Arabic-speaking mothers (living in Greater Western Sydney) on readily available English-language oral health education leaflets that provide advice on maintaining oral health of young children. Second, to evaluate the acceptability of simplified-English and Arabic-translated versions of the existing oral health education leaflets.

## Methods

### Adapting the existing English oral health education leaflets

NSW Health has several resources aimed at improving the oral health of young children. Of these, the two commonly used leaflets include “NSW Messages for a Healthy Mouth” (L1) [34] and “Teach your baby to drink from a cup” (L2) [35]. Both leaflets (L1 and L2) use evidence-based messages to educate parents about maintaining good oral health of their children, risk factors for ECC and prevention of ECC. These leaflets also consist of picture illustrations. The “NSW Messages for a Healthy Mouth” leaflet reinforces the notion that oral health is an integral part of ‘general’ health and comprises of five key messages namely: Eat Well, Drink Well, Clean Well, Play Well and Stay Well. Every message is further explained in written text and pictorials. “Teach your baby to drink from a cup” is a leaflet explaining how to choose the right training cup for a baby and how to teach the baby to drink from a cup at six-months and 6–12-months of age. It also explains what the baby can drink at specified ages in terms of health benefits.

The two original English leaflets were modified specifically for the Arabic-speaking mothers using standardised principles for cultural, linguistic and literacy appropriateness of education materials [36–38]. For example, pictures of traditional Arabic family and specific food-types were used to complement oral health education messages; medical jargons were removed and replaced by simple words, and messages were translated into common Arabic terms (e.g., “buzoogh-al-asnan”) so that a common Arabic-speaking person can understand. The original leaflets were reviewed by a multi-disciplinary team comprising of four project team-members (two English-speaking researchers and two Arabic-speaking researchers) and four lay individuals (mothers of young children) from an Arabic-speaking background. The review team was asked to review the leaflet’s content and identify potential barriers to Arabic community members’ understanding of the health information along with making suggestions to simplify the language, reduce medical jargon and tailor them for cultural appropriateness. Afterwards, the simplified English version of both leaflets (SL1 and SL2) was translated into Arabic language by two Arabic-speaking researchers and two Arabic-speaking lay individuals. The Arabic version of the leaflets (AL1 and AL2) were then back-translated in English by two lay individuals in order to ensure the quality and accuracy of key oral health messages [38]. Revision of the materials was in accordance with the findings at each step.

### Development of the evaluation tools

Two separate semi-structured validated tools [39] were developed by members of our research team to be used for evaluation of the leaflets. The overall purpose of the tools was two fold: firstly, to assess the concept validity and acceptability of oral health advices based on linguistic and literacy skills of participants; and cultural norms. Secondly, to ascertain and improve mismatches in the content, communication and design of simplified English and translated Arabic versions of the leaflets. Each evaluation tool was assessed by four volunteer Arabic community-members who had read the revised leaflets. They were advised to review the tools to ensure that concept validity, acceptability, and comprehension in the leaflets were clearly gauged. Additionally, the reviewers evaluated whether the questions being asked would be understood by Arabic community members. The evaluation tools were revised accordingly.

### Study background

This study was nested within the *Healthy Smiles Healthy Kids (HSHK)* birth cohort study (*n* = 1035), which commenced in 2010 to explore the relationship between early childhood feeding practices and dental caries in preschool children residing in South Western Sydney [1]. Child and Family Health Nurses recruited mother-infant dyads at the first post-natal home visit at four to six weeks [40]. As part of the HSHK study, two leaflets giving oral health advice on taking care of children’s teeth were sent by post to the parents. The two leaflets (L1 and L2) were: “Teach your baby to drink from a cup” and “NSW Messages for a Healthy Mouth”.

### Research design

A qualitative research design was utilised to get a deeper understanding of Arabic-speaking mothers’ perspectives on oral health literacy needs and existing education leaflets. Such qualitative approach was used for two reasons [41]. First, it enabled us to collect in-depth data on the maternal perspectives of original and adapted leaflets. Second, the flexibility of research design provided an opportunity for simultaneous data collection and analysis.

### Sampling

We used a purposive sampling strategy, that is, a sampling technique that enriches the data quality by selecting the subjects strategically and purposefully: a commonly used strategy in qualitative research [42]. The researchers adopted a maximum variation sampling method to attain a variation on dimensions of interest and to identify important patterns to enrich our data quality [41, 43]. Previous studies have shown that a sample of 12–15 interviews is sufficient to reach data saturation, defined as “the point at which additional data does not improve the understanding of the phenomenon under study” [43]. From HSHK birth cohort study, 19 Arabic-speaking mother-infant dyads were selected for a home interview from postcodes ranked as “disadvantaged” according to the 2006 Australian Socio Economic Index for Areas (SEIFA) [44]. Furthermore, the following criteria’s were used to choose mothers thereby ensuring a broader perspective:

- either primiparous or multiparous;
- either married, or living with a partner, or were single;
- came from any Arabic-speaking county or origin;
- came from a range of education levels;
- either employed (skilled/unskilled) or unemployed and/or pensioners.

The mothers fulfilling the selection criteria were invited to participate via a phone call and were then sent an information pack containing a participation information statement and a consent form for this nested study. Mothers who gave consent were selected and approached for the interview.

### In-depth semi-structured interviews

On the day of the interview, mothers were given simplified-English (SL1 and SL2) and Arabic (AL1 and AL2) versions of both the leaflets. Two bilingual researchers (HM and IAS) who had experience in population oral health and qualitative interviewing conducted in-depth interviews in Arabic at the homes of study participants. During the one-hour interview, mothers were asked to compare all six leaflets (L1, L2, SL1, SL2, AL1 and AL2) and identify which version facilitated the best source of oral health information. A semi-structured interview guide (Table 1) derived from our previous qualitative research investigation [45] was used to record their opinions and understanding about the leaflets. Throughout these discussions, participants not only justified their preference for a particular version of the leaflet, but also discussed any issues they had with the leaflets. All interviews were audio recorded, debriefed, and translated from Arabic to English and transcribed *verbatim*.

**Table 1:** Interview guide

1. What are the key messages you got from the leaflet?
2. What made the leaflet easy/difficult to read?
3. Is there anything you were unable to read? If so, what is that you were unable to read?
4. What made the leaflet easy/difficult to understand? Is there anything you were unable to understand?
5. If so, what is that you are unable to understand?
6. Did you need help from anyone?
7. Did the leaflet had any missing information? If so, what was missing?
8. What do you like the most/least about the leaflet?
9. Would you recommend this leaflet for others? Why?
10. Do you have any further suggestions?

### Evaluation of the education materials and data analysis

The evaluation tools were administered by trained bilingual researchers at the interview session in the participants’ language of choice. Data from closed-ended questions were presented as percentages. Responses to open-ended questions were translated by the interviewers and recorded in English. If interviewers were not clear about the meaning of words or phrases used by participants, additional time was taken to clarify.

To improve the rigour and credibility, five researchers were heavily involved in the data analysis, which included debriefing, transcript coding, and interpretation. Following each interview, the researchers reflected on data collection, summarised the main findings and prepared for subsequent interviews. Thematic analysis was undertaken to interpret the main findings of the interview transcripts. This process was conducted in three stages. First, transcripts were analysed line by line; and first-level of coding and common themes was performed by the principal researcher (AA) using NVivo 9 (QSR International, Cambridge, MA, USA). Second, four researchers (NT, IAS, HM, and RI) independently categorised, re-categorised and condensed some of the first-level codes based on overlap or similarity of some responses. They reviewed the transcripts systematically and identified the concepts provided by the participants. The parent codes derived from our analysis included topics such as literacy, comprehension, translations, and culture. The sub-codes included words such as medical terms, native language, interpretation, tailor, target, simple, pictures, layout, and visual. Third, all five researchers reviewed the second-level coding and their corresponding passages through an iterative process, regrouping them into broader themes. All the researchers reached a consensus on any discrepant categorisation through ongoing discussion.

### Ethical considerations

Ethical approval for this study was acquired from the Human Research Ethics Committees of the former Sydney South West Area Health Service, University of Sydney and Western Sydney University.

## Results

All 19 mothers participated in the interviews, a response rate of 100%. All women resided in South Western Sydney, and their children’s ages ranged from 6 months to 18 months. Sixteen out of total 19 mothers were aged between 20 and 40 years and 14 of them had year 12 or lower level of education. In general, mothers with lower levels of education reported that the original leaflets were harder to read and understand compared to the tailored versions of the leaflets. Additional socio-demographic details of participants (namely, number of children, country of birth) are described in Table 2.

**Table 2:** Socio-demographic characteristics of the study participants (*n* = 19)

Characteristic	N
Parity	
Primiparous	8
Multiparous	11
Mother’s age (in years)	
20–29	7
30–39	9
≥ 40	3
Mother’s country of birth	
Egypt	3
Iraq	4
Lebanon	4
Saudi Arabia	2
Syria	3
United Arab Emirates	3
Mother’s level of education	
≤ year 12	14
College or university	5
Mother’s occupation	
Unemployed	11
Unskilled workers	6
Skilled workers	2
Marital status	
Married	15
Single	4

### Evaluation of the simplified English education leaflets (Table 3)

Eighteen participants read the simplified English leaflets. Seventeen of the 18 (94.4%) participants indicated that the simplified English leaflets helped them better understand on how to look after children’s oral health compared to the original English leaflets. Thirteen participants indicated that the original leaflets were more difficult to understand than the simplified leaflets. One participant felt the simplified leaflets were missing information. Interestingly, all participants believed that the simplified leaflets helped them make better health choices for their children’s oral health.

**Table 3:** Simplified English leaflets questionnaire and responses

Did you read the leaflets? Yes n = 18 No n = 1	
Did you find the simplified leaflets helped you to understand how to look after children's oral health? Yes n =17 (94.4%)^a^ No n =1 (5.6%)	
What helped you to understand the simplified leaflets? This is easier to understand I like the pictures Very simplified and attractive	
Did you find the original leaflets were more difficult to understand compared to the simplified English version? Yes n = 13 (72.2%) No n = 5 (27.8%)	
Please tell us what part was difficult? I didn"t understand fizzy drinks The part where the leaflet talks about leaving the kids with a bottle at night	
Did you find the simplified leaflets had any missing information compared to the original leaflets? Yes n = 1 (5.6%) No n = 17 (94.4%)	
What information is specifically missing? It has dot points but is not very detailed, it may be useful only for non-fluent English speakers	
Do the simplified leaflets help you to make better health choices for your child's oral health? Yes n = 18 (100%) No n = 0 (0%)	

^a^Percents are based on the numbers of participants that read the pamphlet (n = 18)

### Evaluation of the translated Arabic education leaflets (Table 4)

Of the 19 participants who read the Arabic leaflets, 16 (84.2%) found it useful to receive the information in their own language. Three participants indicated that they did not find the translations useful particularly because Arabic has different dialects. Seventeen (89.5%) participants indicated that the Arabic leaflets helped them better understand on how to look after children’s oral health. Eighteen of the 19 (94.7%) participants indicated that the Arabic leaflets helped them make better health choices for their children’s oral health. One participant preferred the simplified English leaflets over the Arabic leaflets.

**Table 4:** Translated Arabic leaflets questionnaire and responses

Did you read the leaflets? Yes n = 19 No n = 0	
Was it useful to receive information on child's oral health in Arabic? Yes n = 16 (84.2%)^a^ No n = 3 (15.8%)	
If No, please explain why? Arabic has different dialects, so the translations did not help	
Did you find the Arabic leaflets helped you to understand how to look after children's teeth? Yes n =17 (89.5%) No n = 2 (10.5%)	
What helped you to understand the Arabic leaflets? All of it Dot points and the size of the text	
Do the leaflets help you to make better health choices for your child's oral health? Yes n= 18 (94.7%) No n = 1 (5.3%)	

^a^Percents are based on the numbers of participants that read the pamphlet (n = 19)

### Themes emerged from the qualitative data

The following sections explain the three main themes that emerged from the qualitative data: (1) Preference for Arabic leaflets and Critique in translations, (2) Preference for simplified-English leaflets and its Complimentary reading with Arabic leaflets (3) The importance of visual representation: simple layout and use of pictures**.**

### Theme 1 - preference for Arabic leaflets and critique in translations

A recurrent theme was a sense that oral health promotion materials written in Arabic language were the preferred choice for oral health information. Majority of participants indicated that the leaflets in Arabic language (AL1 and AL2) were the most useful medium of oral health education; and are easier to read and understand compared to the ones in English (L1, L2, SL1 and SL2).

*“It’s always easier reading it in Arabic so I’ll be less picky about the Arabic version than the English one because I can understand it better”.*

*“…I can read Arabic more fluently than English so I get to pick up the information quicker and understand it well. When I read it in English, it took me longer to read and of course there were words that I didn’t understand…”*

Despite an overall preference for the Arabic leaflets, some participants felt that the Arabic translations were inaccurate or confusing, particularly the use of ‘formal Arabic terms’, or Arabic terms used exclusively by those from a small number of Arabic speaking countries.

*“[Translation of ‘sealants’ to ‘protective tooth paint’] this makes it sound like nail polish (laughs). I think maybe just translate it to “protective filling” and then with the help of a picture show it on a tooth.”*

Despite a consensus on the usefulness of leaflets in their native language, interviewees provided valuable critique on the usefulness of Arabic translations. The risks of homogenising cultural groups through certain translation terminologies were highlighted. As suggested by participants, this can be overcome by utilising simple terminology that transcends across all groups, rather than incorporation of culturally specific ‘slang’ or use of formal language.

*“…change “buzoogh al-asnan” (eruption of teeth) to “thuhoor al-asnan” (appearance of teeth). I don’t think the word “buzoogh” (eruption) is that commonly used.”*

*“Word “bozoog” if you can change it to “zohoor”. We are used to the simple language rather than formal Arabic”.*

### Theme 2 – Preference for simplified-English leaflets and its complimentary reading with Arabic leaflets (dual Arabic-English leaflet)

A consensus in participant preference for the simplified English version, rather the original English leaflet was also expressed. The participants expressed difficulty in understanding the medical jargon and technical language used in the original leaflet, which was explained in lay terms in the simplified English leaflets.

*“I think it’s [original English] packed with writing and it may be too much for some mothers to read, especially if they are slow English readers”.*

*“I guess ‘less is more’. If you were at a doctor’s surgery and pick this up to read then you are more likely to finish reading it and get the important bits of information before let’s say the doctor calls you in”.*

The informant perspectives shed light on how the complimentary reading of the Arabic leaflets alongside the simplified English versions facilitated the improvement of the mothers’ English literacy skills. This was due to the simplified English version providing a clearer understanding of the messages depicted in the leaflets. Furthermore, it also enabled mothers to understand medical terminology used in dental clinics and healthcare environments in Australia.

*“If I see a pamphlet in English and Arabic written next to it then I’d definitely pick it up to see what certain words are in English. This would help in improving my English.”*

*“Personally I’d pick up both because for me I’d want to learn more English words and improve my English that way. So this is why I’d do it this way.”*

As reflected in the above testimonials, certain terminologies were more understandable when Arabic to English synonyms were used. Many terms and phrases were not new, but more difficult to understand in the original English version. As asserted by the participants, the simplified English and Arabic versions of the leaflet were complimentary assets to aid the comprehension of key terminology. A very interesting finding emerging from this study was the need of a ‘dual Arabic-English leaflet’, which would assist Arabic mothers to read and interpret the key health messages (written in English) by matching them with Arabic words. They believed that such leaflets would also improve their English vocabulary and reading skills.

*“Can’t you make a dual Arabic-English pamphlet, where each line is translated for those who know a little bit of English but want to learn new English words to improve their vocabulary for when they visit the dentist?”*

Together these results highlighted the importance of tailoring oral health messages to the literacy needs of the target populations. Further, it points to the benefits of this ‘targeted’ approach in not only enhancing the health literacy of disadvantaged communities, but also improving overall English language competency – an essential skill for navigating the vast and complex health system in an English-speaking nation like Australia.

*“English is useful. If you give me both I learn any word I’m missing in the English version… we want to learn these words because we get exposed to it in hospitals”.*

### Theme 3 - the importance of visual representation: Simple layout and use of pictures

A preference for using a ‘dot point’ structure, rather than paragraphs in the leaflets was expressed by mothers. This was attributed to enhanced comprehensibility of key messages and issues covered in the leaflets.

*“I think for non-fluent English speakers it may be easiest to read summarised points rather than full sentences. This is because they will get the message quickly rather than having to read through more text to get the same messages”.*

Additionally, most informants mentioned that oral health education material must include visual images that complement the messages provided in the text in the form of pictures, photos, diagrams or labelling.

*“The colours would also capture the mother’s attention. Adding pictures will definitely help because we can then put “picture to word””.*

Mothers preferred the simple layout of written content to be accompanied by visual images such as diagrams, graphs and pictures that relay the key oral health messages.

*“The pictures let us know that this pamphlet would be talking about things like food (points to food image), caring for children’s teeth. The colours are also bright and give the impression that the pamphlet is about children’s health. The font is also like chalk making it appear to be directed at young children’s health”.*

The perspectives provided by the Arabic-speaking mothers in this study indicate the benefits of simplified information along with visual design of oral health promotion materials. Indeed, the integration of high quality visual material in a simple layout may enhance the understanding of key health messages, and possibly improve the health literacy of disadvantaged groups such as non-English speaking communities.

*“Pictures are always understood by people without having to read much….The pictures are clear and expressive, even if the person didn’t read all the writing they can still get the message clearly by looking at the picture”.*

## Discussion

Oral health literacy is the “degree to which individuals have the capacity to obtain, process and understand basic oral health information and services to make appropriate health decisions” [46]. This study provides insight into the acceptability and need for simplified English and translated versions of commonly available oral health education leaflets to fit the needs of Arabic-speaking mothers with young children in Greater Western Sydney, Australia. In terms of socio-demographic characteristics, mothers with lower levels of education reported that the original leaflets were harder to read and understand compared to the tailored versions of the leaflets. While mothers favoured reading the simplified version over the original English leaflets, the majority favoured the leaflets in their native language. A more preferable choice was having the simplified English version (SL1 and SL2) compliment the Arabic version (AL1 and AL2) in the same document. The understanding of key oral health promotion messages was optimised through the use of a simple layout, simple language and incorporating complimentary visual images. The current findings reaffirm the need to actively engage the target audience in the development process of oral health promotion material in order to enhance the value of the educational materials among ethnic minority groups [15, 16, 47–50].

Input from the nineteen Arabic-speaking mothers in this study stresses that the oral health literacy of ethnic minorities is more likely to be enhanced by presenting health information in a simplified format when accompanied by an informal/conversational writing style and visual images that complement the content. As highlighted by the empirical data in this study, the need to provide leaflets, in the native language, and ‘simplified English’ not only is more likely to enhance oral health literacy, but also has the ability to improve English proficiency and literacy skills overall. This reaffirms the findings of Jones et al. [39] who highlighted the overall value of the simplified educational materials for a Canadian South Asian community. Mothers preferred reading the simplified English leaflet compared to the original English leaflet. This was largely attributed to the simplification of medical jargon and technical terminology in the original format. This key finding mirrors the main opinions expressed by English-speaking, Chinese-speaking and Vietnamese-speaking mothers living in South-Western Sydney [15, 17, 47].

Most notably, a preference for reading the Arabic translated leaflet, alongside the simplified English version provides valuable insight into the role that health education can have in enhancing English literacy. It also highlights the positive effects that well-developed health promotion materials can have in educating the ethnic minority groups regarding Australia’s health system and processes.

The Arabic-speaking mothers interviewed in this study favoured the educational leaflets written in their native language. This was mainly due to greater readability and understanding of the material than what was attained in reading both versions of English leaflets. Although participants preferred the Arabic version, they were also open to receiving oral health education in English, as long as this was provided in simplified, colourful and ‘visually robust’ mediums. Many participants also expressed a preference to read the Arabic in conjunction with simplified English versions to assist with their English literacy skills. The preference for the simplified English version was mainly due to concerns that the medical jargon, lengthy text and complex words made the original English version difficult to read whereas when the language was simplified, or participants substituted their own words, the readability and their understanding of the messages improved. As Kwan [51] suggests, this may occur due to the differences in language structure and grammar, whereby very literal translations from one language to English may lead to confusion, change the intended meaning and even impede understanding. Further, the need to consider different variations and ‘slang’ of certain languages, such as Arabic when producing translational material was highlighted. These findings provide valuable insight on how to produce more appropriate leaflets based on colloquial Arabic idioms, whilst also enhancing the English literacy skills of ethnic minorities concurrently.

In this study, mothers critiqued the English to Arabic translations and suggested using simple terminologies that transcends across all Arabic groups, rather than use of culturally specific ‘slang’ or use of formal language. This is a common finding in research conducted in an Arabic context since Arabic is a diglossic language, i.e. it consists of two different varieties – one High variety that is formal and is mainly written while the Low variety is spoken and is used in daily life [52]. This Low variety of Arabic language differs across all Arabic-speaking countries making a wide array of Arabic dialects. This is of particular significance to public health researchers working with different Arabic-speaking migrants since every region and country represents unique culture and people [53].

The mothers in this study preferred pictures and a simplified layout, suggesting that such format helped them to more rapidly understand the main concepts being communicated. This has also been reported elsewhere [39, 54, 55]. These findings are supported by research in psychology, indicating that humans have a preference for picture-based information, rather than text-based information [56]. Furthermore, the health communication literature [54, 55] suggests that adding pictures to leaflets can significantly improve patient interest, recall, comprehension, and behaviours. This finding is particularly important in English-speaking countries as visual support is commonly used in Teachers of English to Speakers of Other Languages (TESOL) and Teaching English as a Foreign Language (TEFL) classes worldwide [57].

### Strengths of the study

This study had a number of strengths that are worth reporting. Firstly, we used a qualitative approach to get an in-depth understanding of the Arabic-speaking migrants in Greater Western Sydney. The flexibility of the research design gives an opportunity for further investigation if required and fosters simultaneous data collection and analysis [41]. Secondly, we approached 19 Arabic-speaking mothers from the ongoing HSHK study and all agreed to be a part of this nested study, thus achieving a 100% response rate. A sample of 19 research participants was enough to reach data saturation, that is all the dimensions of interest were explored and no new information would have been collected from interviewing more participants [43]. Third, the strong community partnership that our team has established with ethnic minorities through the HSHK study is crucial for the ongoing development of tailored oral health education leaflets. Fourth, some members of the research team in this study were from an Arabic-speaking background that provided a cultural insider aspect to the development of leaflets, interview process and data analysis. Finally, in our research, male researchers interviewed young Arabic mothers; this entails cross-gender research in terms of research positionality. In most instances, men’s status in feminism is still marginalised no matter how much they are committed to women’s problems and concerns [58].

### Limitations of the study

There are several limitations of this study. Firstly, we were not able to assess whether the simplified English and Arabic-translated leaflets were actually able to improve the oral health practices of mothers and children. Secondly, we were not able to record the participating mother’s period of residence in Australia and what impact did that have on their health literacy level. Third, the status and influence of fathers’ oral health literacy on children’s oral health were not explored although they also share the responsibility of maintaining children’s oral health. Fourth, the participants’ level of understanding was measured only by their need for help and their personal description of the difficulty to read the leaflet. Fifth, the original leaflets ((L1 and L2) are readily available in NSW and are used as part of the ongoing HSHK study. Therefore, mothers had access to these leaflets before the interview and they may have taken help to understand the content of the original leaflets. Nonetheless, the simplified and translated versions of the leaflets, which were given on the day of the interview, were deemed more appropriate by the Arabic-speaking mothers. Finally, the use of a small sample of Arabic-speaking mothers living in South Western Sydney limits the generalisation of our findings to the broad range of literacy skills shared amongst other disadvantaged and culturally diverse families.

## Conclusion

The empirical findings of this study brings our attention to the need for ongoing development and distribution of health education material in formats that are readable and useful to potential user groups whose first language is not English. The Arabic mothers felt that existing oral health education leaflets were too complex and they had difficulty understanding the key messages; whereas they were particularly receptive to the Arabic-translated leaflet. Also of interest was the finding that many Arabic mothers would prefer a ‘dual Arabic-English leaflet’ for better understanding and improving their English literacy skills. This study suggests the need to tailor information to the language and literacy level of the target audience, along with the need for a more visual and simple layout to optimise readability and understanding. Proactive engagement of the target community during the process of designing oral health promotion material is critical to ensure cultural and linguistic appropriateness of health education materials. The process of simplifying the content of health education leaflets may be useful to other researchers and policy makers who wish to develop leaflets for people with limited literacy skills.

## Abbreviations

ABS: Australian bureau of statistics
AIHW: Australian institute of health and welfare
ECC: Early childhood caries
NHMRC: National health and medical research council
NSW: New South Wales
SEIFA: Socio economic index for areas
TEFL: Teaching english as a foreign language
TESOL: Teachers of english to speakers of other languages
